# Health-related quality of life of children with Williams syndrome and caregivers in China

**DOI:** 10.3389/fpubh.2023.1177317

**Published:** 2023-06-09

**Authors:** Weijun Chen, Lidan Sun, Xinyu He, Ziqiao Li, Chai Ji, Fangfang Li, Jiyang Shen, Tianxin Pan, Xuejing Jin, Yusang Dong, Lidan Hu, Chaochun Zou, Guannan Bai

**Affiliations:** ^1^Department of Child Health Care, Children's Hospital, Zhejiang University School of Medicine, National Clinical Research Center for Child Health, Hangzhou, Zhejiang, China; ^2^Children's Hospital, Zhejiang University School of Medicine, National Children's Regional Medical Center, National Clinical Research Center for Child Health, Hangzhou, Zhejiang, China; ^3^Health Economics Unit, Centre for Health Policy, Melbourne School of Population and Global Health, The University of Melbourne, Melbourne, VIC, Australia; ^4^Centre for Evidence-Based Chinese Medicine, Beijing University of Chinese Medicine, Beijing, China; ^5^Department of Endocrinology, Children's Hospital, Zhejiang University School of Medicine, National Children's Regional Medical Center, National Clinical Research Center for Child Health, Hangzhou, Zhejiang, China

**Keywords:** Williams syndrome, quality of life, PedsQL, children, family impact

## Abstract

**Introduction:**

Williams syndrome (WS) is a rare genetic disorder that impacts multiple systems and may cause developmental delays. These medical and developmental issues impose a heavy burden on affected children and their families. However, there was no study on children’s health-related quality of life (HRQoL) with WS and only two studies about family quality of life globally. Therefore, the primary purpose of this study was to assess the HRQoL of children with WS and their caregivers in China, and the secondary purpose was to identify the potential determinants of children’s and caregivers’ HRQoL.

**Methods:**

In total, 101 children and caregivers were included. We applied the proxy-reported PedsQL 4.0 Generic Core Module (PedsQL GCM) and PedsQL 3.0 Family Impact Module (FIM) to measure the HRQoL of children and caregivers. Additionally, we collected information on a comprehensive set of social demographic and clinical characteristics. Differences in HRQoL scores across subgroups were assessed by two-independent-samples *t*-tests, one-way ANOVA, and *post hoc* tests. We also calculated effect sizes to indicate clinical relevance. Multivariate linear regression models were applied to assess the potential determinants of HRQoL.

**Results:**

We found that the HRQoL of children with WS and their caregivers was dramatically worse than the norm average scores of the healthy controls of children published in previous studies. Paternal educational level, household income, and the perceived financial burden significantly influenced the HRQoL of both children and families (*p*-values < 0.05). Multivariate linear regression analysis showed that the perceived financial burden was independently associated with family quality of life (*p*-values < 0.05)., and the presence of sleeping problem was independently associated with children’s HRQoL (*p*-value = 0.01).

**Conclusion:**

We call for attention from policymakers and other stakeholders on the health status and well-being of children with WS and their families. Supports are needed to relieve psychosocial distress and financial burden.

## Highlights

The media use some descriptions to introduce children with Williams syndrome (WS), such as “friendly to extreme,” “the opposite of autism,” and “happy smiling face”. Are these children really happy? There has been no data on children’s health-related quality of life (HRQoL) with WS. The present study has filled this knowledge gap by providing rich data on the HRQoL of a relatively large sample of Chinese children with WS and their caregivers using reliable and validated instruments. Notably, this study highlighted the significantly worse HRQoL of both children and caregivers than the healthy references. In addition, we must address that the low socioeconomic status indicated by parents’ education, household income, and the subjective perception of financial burden impair multiple domains of HRQoL of children and caregivers, highlighting the importance of social support for families with WS.

## 1. Introduction

Williams syndrome (WS) or Williams-Beuren Syndrome (WBS) is a rare genetic disorder affecting approximately one in every 20,000 live births, with both genders equally involved (1). The prevalence was reported as 1/23,500 in Hong Kong and there is no such data in the mainland China (2). It is a complicated neurodevelopmental disorder caused by a deletion of around 26 genes on chromosome 7q11.23, and these genes are involved in the development of multiple systems (3). WS is found in all racial and ethnic groups. Individuals with WS often display distinctive facial features, including a broad forehead, full cheeks, and a small, upturned nose, known as a “pixie face.” Individuals with WS usually have an extremely friendly and outgoing personality and a high degree of verbal ability. In addition, WS is associated with many medical characteristics, such as congenital heart disease, connective tissue abnormalities, and growth deficiency (4). Most WS children may experience developmental delays in early childhood and mild to moderate intellectual disability at school age (5). These medical and developmental issues greatly distress families and individuals with WS, significantly impacting the quality of life of affected children and their families.

Quality of life is a significant concept and a principal endpoint for research and practice in public health and medicine (6). According to the World Health Organization Quality of Life Assessment Group, it is defined as “an individual’s perception of their position in the life in the context of the culture in which they live and in relation to their goals, expectations, standards and concerns” (7). Health-related quality of life (HRQoL) is a term referring to the health aspects of quality of life and is generally considered to reflect the impact of disease and treatment on disability and daily functioning; it has also been considered to reflect the impact of perceived health on an individual’s ability to live a fulfilling life (8). HRQoL is a subjective and multiple-dimensional concept measuring physical, mental, and social health domains.

Children with WS and their families may face significant challenges to their physical, mental, and social health or well-being. Due to physical, developmental, and behavioral issues, children with WS may require ongoing medical care and treatment. Caregivers may also be exposed to significant emotional, financial, and practical distress when they have to provide ongoing care for their loved ones suffering from WS-related health problems. Based on the literature review, we only found two studies about quality of life-related to WS. Sepúlveda et al. in Spain found that the degree and the presence of intellectual disability did not homogeneously influence the family quality of life, and many variables such as material well-being and interpersonal development could affect the family quality of life (9). Pereira et al. in Brazil found that paternal education, children’s cardiopathy, and autonomy in activities of daily living had an impact on the overall family quality of life (10). The studies mentioned above are about family quality of life, and the sample sizes were relatively small. To our knowledge, there was no study on HRQoL of children with WS. This might be explained by the fact that due to the intellectual and cognitive impairments, children with WS cannot evaluate their HRQoL by themselves, so proxy-reported HRQoL is an alternative. In addition, few instruments measure the HRQoL of pediatric patients with a broad age range, for instance, from infants to adolescents. The Pediatric Quality of Life Inventory (PedsQL), developed by Varni et al. in the United States, may be an appropriate measurement to evaluate the HRQoL of healthy and sick children at different ages (11, 12).

Therefore, the primary purpose of this study was to assess the HRQoL of children with WS and their caregivers in China, and the secondary purpose was to identify the potential determinants of children’s and caregivers’ HRQoL. Our study will benefit from a better understanding of the impacts of WS on children and their families and provide solid evidence for policymakers and other stakeholders, which eventually improves the HRQoL of WS patients.

## 2. Methods

### 2.1. Study design and participants

The present study was embedded in the cohort of pediatric patients with WS in China. Since 2009, we have established a WS cohort including over 300 patients who visited the Department of Child Health Care in the Children’s Hospital affiliated with Zhejiang University School of Medicine in Hangzhou, China. In 2014, we launched a free Multi-Disciplinary Treatment (MDT) program. To follow up with these families, we had an online patient support group via WeChat (a Chinese social media application) and follow-up by phone. From November 2022 to February 2023, we conducted a questionnaire-based survey among 103 WS patients and their families. Included families were required to meet the two criteria: (1) their child was diagnosed as WS; (2) the age range was from 2 to 18 years. Patients whose caregivers were unwilling to fill in the questionnaire or share the clinical data were excluded from the study. In total, 101 patients and their caregivers completed the questionnaire and were included in analyses. Experienced experts developed the survey questionnaire with research or practice backgrounds in public health, patient-reported outcome measures, and pediatric clinicians in neurodevelopmental and behavioral disorders and rare diseases. The questionnaire includes four parts: social demographic information, clinical information, caregiver’s HRQoL, and proxy-reported HRQoL of children. Several rounds of discussion were organized. A pre-test survey was conducted among 12 parents of children with WS aged from 2 to 18 years, followed by a brief interview by phone to collect suggestions from caregivers. The final version questionnaire was revised accordingly by experts to ensure that this questionnaire was easy to understand and feasible for the online survey, reliable, and validated to measure the HRQoL of children and caregivers. The electronic questionnaire was made by the Wenjuanxing application (a survey platform) that generated a QR code for distribution in the WeChat group, i.e., the online WS patients/families peer-support group. Following the principle of voluntary participation, the study questionnaire was sent by a website to the caregivers with detailed information about the purpose and methods of this study. All eligible participants provided electronically signed consent forms before filling in the questionnaire. If caregivers had any questions, they could contact a research assistant who was a physician for further instruction.

The present study was conducted according to the Declaration of Helsinki and was approved by the Ethics Committee of the Children’s Hospital of Zhejiang University School of Medicine (2019-IRB-122).

### 2.2. Measurements of general characteristics

Information on social demographic characteristics, including child’s age, gender, maternal/paternal educational level, marital status, parents living together, household income, residence (urban/rural areas), insurance, cost related to WS treatment, and perceived financial burden due to WS was collected via questionnaire. Education was categorized into three subsequent levels based on the Chinese Standard Classification of Education: high level is a bachelor’s degree or above; middle level includes high school, technical school, vocational secondary school, and vocational high school; and low level refers to no education, primary school or middle school. Living together was measured by a single question, “Do the child’s parents live together” with three options (always, sometimes, and never). The perceived financial burden was measured by the question, “How much burden does the child’s treatment place on the family?” with three options (easily affordable, affordable, hard to afford).

### 2.3. Measurements of clinical characteristics

We also collected a comprehensive set of clinical variables by a questionnaire including the age of onset of the disease, age of diagnosis, genetic test, rehabilitation, disorders at birth, congenital heart disease and surgery, symptoms of attention deficit and hyperactivity, the presence of health conditions of visual system/endocrine system, /digestive system /urinary system conditions, repeated respiratory tract infection, inguinal hernia, and sleeping problems.

### 2.4. Health-related quality of life

We applied the proxy-reported PedsQL 4.0 Generic Core Module (PedsQL GCM) and PedsQL 3.0 Family Impact Module (FIM) to measure the HRQOL of children and caregivers. Both instruments were validated in China with good reliability and validity (13, 14). The proxy-reported PedsQL 4.0 GCM is administrated to measure HRQOL in children and adolescents ages 2–18 years in the past month. It contains 23 items and four scales, i.e., physiological (eight items), emotional (five items), social (five items), and school functioning (five items). It is widely used for healthy children in communities and schools and can distinguish pediatric disease patients from healthy controls (15). The PedsQL 3.0 FIM was used to evaluate the impact of WS on the caregiver’s QoL and family function in the past month. It contains 28 items and six scales, i.e., physical functioning (six items), emotional functioning (five items), social functioning (four items), cognition functioning (four items), communication (three items), worry (five items), daily activity (three items) and family relationships (five items) (13). Both instruments were filled by caregivers. In the present study, the Cronbach’s alpha for PedsQL 3.0 FIM is 0.977 and for GCM is 0.907. The Cronbach’s alpha coefficients for each domain of PedsQL 3.0 FIM were 0.938 (physical functioning), 0.941 (emotional functioning), 0.896 (social functioning), 0.959 (cognition functioning), 0.859 (communication), 0.906 (worry), 0.877 (daily activity) and 0.959 (family relationships), respectively. The Cronbach’s alpha coefficients for each domain of PedsQL 4.0 GCM were 0.859 (physiological functioning), 0.854 (emotional functioning), 0.880 (social functioning), and 0.762 (school functioning), respectively.

The scoring algorithm of PedsQL 4.0 GCM and PedsQL 3.0 FIM is similar. Response choices were provided on a 0 to 4 scale (0 = never a problem, 1 = seldom a problem, 2 = sometimes a problem, 3 = often a problem, and 4 = always a problem). Items are linearly transformed to a 0–100 scale (0 = 0, 1 = 25, 2 = 50, 3 = 75, 4 = 100). Scale scores and total scores are computed as the sum of the items divided by the number of items answered that accounts for missing data. A higher score indicates better HRQoL.

### 2.5. Statistical analysis

Firstly, we applied descriptive analysis to calculate means and standard deviations (SDs) for continuous variables and numbers and percentages for categorical variables. Secondly, we compared the averages of the total score and the scale scores of both PedsQL 4.0 GCM and PedsQL 3.0 FIM with those norm average scores of the healthy controls of children that were reported in the published literature (14, 16). Thirdly, we used the two-independent-samples t-tests and one-way ANOVA to assess the differences in mean scores of PedsQL 4.0 GCM and PedsQL 3.0 FIM across groups regarding social demographic and clinical characteristics. In addition, regarding variables with three or more categories, the *post hoc* test was adopted to evaluate the pairwise differences in the averages of HRQoL total and scale scores between two specific subgroups. Tukey HSD correction was used for multi-group differences comparison. Fourth, we selected variables with statistical significance in the last step and included these variables in the multivariate linear regression models; the outcomes were the total score of PedsQL 4.0 GCM and 3.0 FIM. A *p* < 0.05 was used to indicate the statistical significance of all two-sided tests. Despite of statistical significance, we also evaluated the clinical relevance using Cohen’s effect size (Cohen’s *d*), that was calculated as the absolute value of the difference in average scores divided by the largest SD and was interpreted as follows: 0.2 ≤ d < 0.5, small difference; 0.5 ≤ d < 0.8, moderate difference; and d ≥ 0.8, large difference (17). All statistical analyses were conducted using the R software program, version 4.1.

## 3. Results

### 3.1. General characteristics of the study population

Table 1 presents the sociodemographic characteristics of the study population. The average age of children was 6.35 years, and 65.3% were boys. 47.5% of children had siblings. Mothers filled out 90.1% of the questionnaire. 73.3% of mothers and 70.3% of fathers had a high educational level, i.e., a bachelor’s degree or above. 95% of parents were married, and. 86.1% lived together. 18.8% of households had three or fewer family members living together, 34.7% with four, 24.8% with five, and 21.8% with six or more members. 65.3% of families lived in urban areas. Regarding the annual household income, 31.7% of families earned less than 14,500 US dollars per year, 36.6% earned 14,500 to 36,300 US dollars, 20.8% earned 36,300 to 72,700 US dollars, and 10.9% earned more than 72,700 US dollars. 90.1% of families reported spending less than 14,500 US dollars per year on WS-related treatment, and the cost of 9.9% was between 14,500 to 36,300 US dollars. 79.2% of children with WS had health insurance, and 25.7% received social benefits/bonuses/subsidies. Regarding the perceived financial burden due to taking care of a child with WS, 33.7% considered it a heavy burden they could hardly afford, while 7.9% thought it was easily affordable. 23.8% of children went to a special school or rehabilitation school.

**Table 1:** General characteristics of children with Williams syndrome (*N* = 101).

Variables	Values^*^
Age [mean (SD)]	6.35 (3.11)
**Age group**	
2–4 years	39 (38.6)
5–7 years	33 (32.7)
8–12 years	24 (23.8)
13–18 years	5 (5.0)
**Gender**	
Boy	66 (65.3)
Girl	35 (34.7)
**The caregiver who filled in the questionnaire**	
Mother	91 (90.1)
Father and others	10 (9.9)
**Paternal educational level**	
Low	14 (13.9)
Middle	16 (15.8)
High	71 (70.3)
**Maternal educational level**	
Low	13 (12.9)
Middle	14 (13.9)
High	74 (73.3)
**Parental marital status**	
Married	96 (95.0)
Divorced or widowed	5 (5.0)
**Living together**	
Always	87 (86.1)
Sometimes	10 (9.9)
Never	4 (4.0)
Having siblings, Yes	48(47.5)
**Number of family members living in the same household**	
Three or less	19 (18.8)
Four	35 (34.7)
Five	25 (24.8)
Six and more	22 (21.8)
**Annual household income (US dollars)**	
Less than 14,500	32 (31.7)
14,500 to 36,300	37 (36.6)
36,300 to 72,700	21 (20.8)
More than 72,700	11 (10.9)
**Residence**	
Urban	66 (65.3)
Town or rural	35 (34.7)
**Health insurance**	
No	21 (20.8)
Yes	80 (79.2)
**Receiving social benefits/bonuses/subsidies**	
No	75 (74.3)
Yes	26 (25.7)
**Cost related to WS treatment per year (US dollars)**	
Less than 14,500	91 (90.1)
14,500 to 36,300	10 (9.9)
**Perceived financial burden**	
Easily Affordable	8 (7.9)
Affordable	59 (58.4)
Hard to afford	34 (33.7)
**School type**	
Normal school	51 (50.5)
Special school/Rehabilitation school	24 (23.8)
Not go to school yet	13 (12.9)
Others	13 (12.9)

* This table presents means, standard deviations, numbers, and percentages. Regarding the educational level, low means no education, primary school or middle school; middle level means high school, technical school, vocational secondary school, and vocational high school; high level means bachelor’s degree or above according to the Chinese Standard Classification of Education.

### 3.2. Clinical characteristics of children with WS

Clinical characteristics of children with Williams syndrome are summarized in Table 2. The average age of disease onset was 0.82 years, and the average age of diagnosis was 1.69 years. 90.1% of children got a genetic test. More specifically, 54.4% had Chromosomal Microarray Analysis (CMA); 15.8% had Whole Exome Sequencing (WES); 5.9% had multiplex ligation-dependent probe amplification (MLPA), 7.9% fluorescence *in situ* hybridization (FISH), and 14.9% whole genome sequencing (WGS). 77.2% of the children had diseases or health problems at birth, 62.4% had congenital heart disease (CHD) with no surgery, and 19.8% had CHD and received surgery. 41.6% presented with symptoms of attention deficit and hyperactivity; 28.7% had health conditions of the visual system; 56.4% had **e**ndocrine system diseases; 27.7% had inguinal hernia; 23.8% got repeated respiratory tract infection; 24.8% had digestive system diseases, and 7.9% had urinary system diseases.37.6% of the children had sleeping problems.

**Table 2:** Clinical characteristics of children with Williams syndrome (*N* = 101).

Clinical characteristic	Values*
Age of onset, mean (SD), years	0.82 (1.15)
Age of diagnosis, mean (SD), years	1.69 (2.26)
Having a genetic test, yes	91 (90.1)
Chromosomal Microarray Analysis (CMA), Yes	55 (54.5)
Whole Exome Sequencing (WES)	16 (15.8)
Multiplex ligation-dependent probe amplification (MLPA)	6 (5.9)
Fluorescence *in situ* hybridization (FISH)	8 (7.9)
Whole genome sequencing (WGS)	15 (14.9)
Getting rehabilitation, yes	60 (59.4)
Diseases at birth, yes	78 (77.2)
Congenital heart disease and surgery	
No	18 (17.8)
Having CHD but no surgery	63 (62.4)
Having CHD and surgery, receiving	20 (19.8)
Presence of attention deficit and hyperactivity, yes	42 (41.6)
Visual system conditions (%)	
Normal	17 (16.8)
Abnormal	29 (28.7)
Not tested	55 (54.5)
Endocrinologic system conditions, yes	57 (56.4)
Inguinal hernia	
No	51 (50.5)
Yes	28 (27.7)
Not tested	22 (21.8)
Repeated respiratory tract infection, yes	24 (23.8)
Digestive system conditions, yes	25 (24.8)
Urinary system conditions	
No	55 (54.5)
Yes	8 (7.9)
Not tested	38 (37.6)
Sleeping problems, yes	38 (37.6)

* This table presents means, standard deviations, numbers, and percentages.

### 3.3. Comparing the mean scores of PedsQL FIM and PedsQL GCM with the norm data

Table 3 presents the mean values and standard deviations of scale scores and total scores of PedsQL 3.0 FIM and PedsQL 4.0 GCM measured in patients with Williams syndrome in our study and those of children as healthy controls extracted from the existing published literature (16, 17). Regarding PedsQL3.0 FIM, the averages of scale scores and the total score were significantly lower than the averages of the healthy controls (all *p* values <0.001). The range of effect size was from 0.62 to 1.54. Regarding PedsQL 4.0 GCM scale scores and total scores, the means were also significantly lower than those of the healthy controls (all *p*-values < 0.001). The range of effect size was from 0.88 to 2.03.

**Table 3:** Comparing means of scale scores and total scores of PedsQL FIM and PedsQL GCM between patients with Williams syndrome and the healthy controls*.

	Williams syndrome	Healthy controls	*t* value	*p*-value	Effect size (*d*)
**PedsQL FIM** ^**a**^					
Physical functioning	58.26 ± 24.52	81.79 ± 15.34	−8.910	<0.001	0.96
Emotional functioning	46.05 ± 25.23	79.98 ± 16.92	−11.969	<0.001	1.34
Social functioning	53.18 ± 24.86	83.48 ± 22.83	−8.717	<0.001	1.22
Cognitive functioning	61.84 ± 25.65	77.80 ± 18.73	−5.241	<0.001	0.62
Communication	50.58 ± 28.60	86.53 ± 16.01	−12.430	<0.001	1.26
Worry	38.95 ± 25.44	78.03 ± 19.52	−12.513	<0.001	1.54
Daily activities	51.32 ± 21.98	73.31 ± 20.54	−7.061	<0.001	1.00
Family relationships	64.91 ± 22.79	79.69 ± 18.23	−5.132	<0.001	0.65
Total score	53.14 ± 19.92	80.07 ± 13.98	−11.688	<0.001	1.35
**PedsQL GCM** ^**b**^					
Physical functioning	59.56 ± 20.69	82.18 ± 17.6	−10.579	<0.001	1.09
Emotional functioning	57.77 ± 21.75	76.90 ± 16.6	−9.129	<0.001	0.88
Social functioning	39.90 ± 22.35	85.33 ± 15.4	−22.429	<0.001	2.03
School functioning	36.82 ± 26.48	78.03 ± 16.5	−18.146	<0.001	1.56
Total score	50.77 ± 16.59	80.74 ± 16.9	−15.380	<0.001	1.77

* Mean scores and standard deviations (SDs) of scale scores and total scores of PedsQL 3.0 FIM and PedsQL 4.0 GCM of the healthy controls are from the published literature (14, 16).

a The sample size of the healthy control group for norm values of PedsQL 3.0 FIM is 210, and we only compared the means and SDs of children aged 5–12 years in our study with the children at the same age in the literature (16).

b The sample size of the healthy control group for norm values of PedsQL 4.0 GCM is 284.

### 3.4. Differences in the mean scores of PedsQL FIM domains and the mean of total score across certain group

Table 4 presents differences in the mean values of PedsQL 3.0 FIM scales and total scores across certain groups. We showed the variables with at least one statistically significant difference (*p-*value < 0.05) in terms of mean values of PedsQL 3.0 FIM eight scales and total scores. Specifically, boys had lower mean scores in the scale of physical functioning (*p* = 0.007), emotional functioning (*p* = 0.041), social functioning (*p* = 0.029), communication (*p* = 0.013) and worry (*p* = 0.021), as well as the total score (*p* = 0.012). Regarding paternal high educational level, the mean scores were relatively higher in physical functioning (*p* = 0.007), social functioning (*p* = 0.034), communication (*p* = 0.005), family relationships (*p* = 0.010) scales, as well as total scores (*p* = 0.005). The mean scores for maternal high educational level were only higher on the cognitive function scale (*p* = 0.023). The mean scores on physical and cognitive functioning scales were relatively low when parents sometimes lived together (*p* = 0.028 and *p* = 0.022). Caregivers whose residences were in urban areas reported higher mean scores in physical functioning (*p* = 0.040), social functioning (*p* = 0.027), and cognitive functioning (*p* = 0.030) scales compared with children living in rural areas. Relatively higher mean scores of all scales and the total score were observed across high to low annual household income subgroups (all *p*-values < 0.001). Caregivers whose children had diseases at birth had higher mean scores in physical functioning (*p* = 0.014), emotional functioning (*p* = 0.005), and communication (*p* = 0.014) scales, as well as for the total score (*p* = 0.037). The mean scores were lower in the family relationships scale of PedsQL FIM (*p* = 0.010) for families who reported their children with sleeping problems. Regarding the variables with three or more categories, we further presented the results of pairwise differences by using the *post hoc* test to show the exact distinction between two subgroups (see Supplementary Table S1). Effect sizes of the differences mentioned above presented in Table 4 are shown in Supplementary Table S2. Additionally, we presented the variables without any statistically significant differences (*p*-value > 0.05) in terms of mean values of PedsQL 3.0 FIM eight scales and total scores in Supplementary Table S3.

**Table 4:** Means and standard deviations of PedsQL 3.0 FIM total score and scale scores (*n* = 101).

	Physical functioning	Emotional functioning	Social functioning	Cognitive functioning	Communication	Worry	Daily activities	Family relationships	Total score
**Gender**									
Boy (*n* = 66)	53.60 (25.44)	44.32 (25.05)	51.61 (25.62)	58.03 (25.49)	46.59 (29.05)	33.26 (25.11)	50.38 (24.70)	60.91 (23.57)	49.84 (21.15)
Girl (*n* = 35)	68.10 (25.06)	55.43 (26.88)	63.57 (26.14)	67.71 (24.68)	61.43 (26.44)	45.86 (26.75)	59.29 (25.23)	68.43 (28.12)	61.23 (21.54)
*p*-value	0.007	0.041	0.029	0.069	0.013	0.021	0.090	0.157	0.012
**Paternal educational level**									
Low (*n* = 14)	39.58 (18.11)	38.93 (22.12)	39.73 (26.59)	38.21 (16.01)	32.14 (23.31)	26.07 (19.03)	40.48 (22.85)	45.00 (24.65)	37.52 (18.88)
Middle (*n* = 16)	55.99 (24.48)	40.62 (24.07)	53.52 (24.26)	59.38 (21.36)	44.27 (29.14)	33.12 (24.89)	56.25 (25.73)	63.44 (19.21)	50.82 (19.28)
High (*n* = 71)	62.97 (26.31)	51.69 (26.77)	59.42 (25.79)	66.41 (25.51)	57.28 (28.13)	40.92 (27.25)	55.40 (24.96)	67.18 (25.38)	57.66 (21.61)
*p*-value	0.007	0.111	0.034	<0.001	0.005	0.116	0.112	0.010	0.005
**Maternal educational level**									
Low (*n* = 13)	49.68 (26.49)	41.92 (20.37)	49.04 (26.50)	48.85 (17.81)	42.95 (28.84)	36.92 (27.95)	46.15 (25.60)	51.92 (26.50)	45.93 (21.72)
Middle (*n* = 14)	51.79 (30.17)	37.50 (25.85)	45.98 (18.28)	51.07 (23.87)	45.24 (25.88)	32.50 (22.51)	53.57 (20.34)	68.93 (20.86)	48.32 (18.09)
High (*n* = 74)	61.49 (25.02)	51.28 (26.61)	58.78 (27.15)	65.54 (25.93)	54.50 (29.33)	38.72 (26.82)	54.73 (25.92)	64.53 (25.63)	56.20 (22.29)
*p*-value	0.186	0.126	0.153	0.023	0.277	0.719	0.529	0.177	0.179
**Living together**									
Always (*n* = 87)	60.20 (25.86)	49.89 (26.80)	55.96 (26.27)	63.85 (25.09)	53.35 (29.54)	39.60 (26.91)	54.50 (25.25)	65.63 (24.13)	55.37 (21.90)
Sometimes (*n* = 10)	39.17 (17.15)	35.50 (18.33)	48.75 (22.20)	40.50 (18.63)	35.83 (17.59)	24.00 (16.96)	45.00 (14.80)	53.00 (29.08)	40.22 (16.60)
Never (*n* = 4)	72.92 (33.07)	42.50 (21.79)	68.75 (37.50)	60.00 (31.36)	56.25 (31.46)	28.75 (22.87)	52.08 (42.70)	43.75 (34.25)	53.12 (25.94)
*p*-value	0.028	0.234	0.434	0.022	0.184	0.162	0.528	0.092	0.115
**Numbers of family members living in the same household**									
Three or less (*n* = 19)	52.85 (25.91)	33.42 (23.46)	50.33 (24.25)	49.47 (23.09)	42.11 (26.86)	29.74 (25.03)	41.23 (22.99)	51.58 (24.84)	43.84 (19.83)
Four (*n* = 35)	62.14 (23.79)	47.71 (21.47)	57.14 (22.80)	69.43 (23.10)	52.62 (27.92)	37.00 (26.60)	55.71 (23.81)	69.86 (23.99)	56.45 (18.83)
Five (*n* = 25)	50.50 (28.52)	45.60 (31.37)	52.50 (31.41)	53.00 (26.85)	49.33 (29.06)	35.80 (25.36)	52.00 (28.59)	60.20 (29.28)	49.87 (25.31)
Six and more (*n* = 22)	67.23 (24.91)	64.55 (20.47)	61.93 (27.20)	68.41 (24.17)	61.36 (30.81)	47.50 (26.49)	62.12 (21.78)	67.50 (19.87)	62.58 (20.82)
*p-*value	0.093	0.001	0.481	0.006	0.192	0.174	0.055	0.058	0.029
**Annual household income (US dollars)**									
Less than 14,500 (*n* = 32)	41.41 (22.92)	28.75 (19.01)	38.09 (23.57)	43.12 (22.10)	30.99 (21.09)	23.12 (17.72)	41.15 (23.94)	48.91 (25.93)	36.94 (15.61)
14,500 to 36,300 (*n* = 37)	61.04 (23.53)	56.76 (24.78)	57.94 (21.58)	67.03 (21.42)	56.98 (28.70)	39.86 (26.91)	52.25 (21.49)	66.08 (22.08)	57.24 (19.38)
36,300 to 72,700 (*n* = 21)	71.23 (23.01)	58.57 (22.92)	73.51 (22.44)	68.57 (24.91)	62.30 (24.95)	49.05 (25.48)	67.86 (24.62)	71.67 (21.76)	65.34 (20.60)
More than 72,700 (*n* = 11)	76.52 (22.07)	55.91 (25.77)	65.91 (26.86)	81.82 (18.74)	74.24 (22.50)	50.45 (29.45)	65.91 (23.70)	81.82 (20.65)	69.07 (18.33)
*p*-value	<0.001	<0.001	<0.001	<0.001	<0.001	<0.001	<0.001	<0.001	<0.001
**Residence**									
Urban (*n* = 66)	62.50 (26.90)	48.03 (26.77)	59.94 (25.73)	65.38 (26.29)	54.92 (28.98)	40.76 (27.02)	55.30 (26.34)	65.08 (26.28)	56.49 (22.27)
Town or rural (*n* = 35)	51.31 (23.22)	48.43 (25.20)	47.86 (25.90)	53.86 (22.46)	45.71 (28.25)	31.71 (24.01)	50.00 (22.60)	60.57 (23.60)	48.68 (20.43)
*p*-value	0.040	0.942	0.027	0.030	0.128	0.100	0.315	0.398	0.088
**Perceived financial burden**									
Easily Affordable (*n* = 8)	86.46 (17.64)	68.12 (16.02)	78.91 (20.85)	90.00 (14.39)	77.08 (23.04)	56.25 (24.89)	78.12 (21.79)	79.38 (16.78)	76.79 (15.72)
Affordable (*n* = 59)	66.24 (21.92)	55.85 (23.75)	63.24 (22.30)	67.29 (21.88)	60.31 (26.05)	44.32 (25.74)	59.60 (22.15)	69.75 (21.42)	60.83 (18.17)
Hard to afford (*n* = 34)	38.85 (21.51)	30.15 (22.00)	37.32 (23.46)	44.41 (23.02)	30.88 (22.24)	21.62 (19.10)	37.01 (21.34)	48.97 (27.07)	36.15 (16.70)
*p*-value	<0.001	<0.001	<0.001	<0.001	<0.001	<0.001	<0.001	<0.001	<0.001
**Having diseases at birth**									
No (*n* = 23)	46.92 (25.91)	35.00 (22.21)	48.64 (28.39)	54.13 (24.06)	38.77 (24.70)	30.00 (20.50)	46.38 (21.88)	63.91 (24.82)	45.47 (19.92)
Yes (*n* = 78)	62.07 (25.33)	52.05 (26.03)	57.85 (25.47)	63.53 (25.69)	55.56 (29.11)	39.87 (27.43)	55.56 (25.75)	63.40 (25.67)	56.24 (21.93)
*p*-value	0.014	0.005	0.141	0.121	0.014	0.113	0.124	0.932	0.037
**Having sleeping problems**									
No (*n* = 63)	61.97 (25.97)	50.24 (25.71)	57.54 (25.60)	62.54 (25.87)	55.42 (28.76)	40.40 (26.67)	57.01 (24.47)	68.49 (22.98)	56.70 (20.99)
Yes (*n* = 38)	53.07 (25.75)	44.74 (26.76)	52.80 (27.52)	59.47 (25.14)	45.61 (28.52)	33.03 (25.22)	47.59 (25.40)	55.26 (27.21)	48.95 (22.71)
*p*-value	0.097	0.307	0.383	0.561	0.099	0.173	0.068	0.010	0.084

* Values presented in this table are means and standard deviations. Regarding the educational level, low means no education, primary school, or middle school; middle level means high school, technical school, vocational secondary school and vocational high school; high level means bachelor’s degree or above according to the Chinese Standard Classification of Education.

### 3.5. Differences in mean scores of PedsQL GCM domains and the mean of total score across certain groups

Table 5 presents differences in the means of PedsQL 4.0 GCM total score and scale scores across certain groups. We presented the variables with at least one statistically significant difference (*p-*value < 0.05) regarding means of total score and scale scores. More specifically, the mean score of physical functioning was observed to be relatively low in children whose families can hardly afford the cost of treatment (*p* = 0.001) and children who had sleeping problems (*p* = 0.013). Regarding the average score of emotional functioning, we observed lower scores in children whose fathers had low education (*p* = 0.012), children whose families had low annual household income (*p* = 0.049) and could hardly afford the cost of treatment (*p* = 0.022), as well as those who had a sleeping problem (*p* = 0.005). The mean score of social functioning was relatively low in children who had symptoms of attention deficit and hyperactivity (*p* = 0.032). Regarding the average score of school functioning, we observed mean scores were significantly different across school types (*p* < 0.001) and was lower in children who had a sleeping problem (*p* = 0.011). The mean of the total score was relatively low in children whose fathers had low education (*p* = 0.023) and whose family could hardly afford the cost of treatment (*p* = 0.002), and children who had sleeping problem (*p* = 0.004); it also was significantly different across household income categories (*p* = 0.044). Regarding the variables with three or more categories, we further presented the results of pairwise differences by using the *post hoc* test to show the exact difference between two subgroups (see Supplementary Table S4). Effect sizes of the differences as mentioned above presented in Table 5 are shown in Supplementary Table S5. Additionally, we presented the variables without any statistically significant differences (*p*-value > 0.05) in terms of mean values of PedsQL 4.0 GCM scales and total scores in Supplementary Table S6.

**Table 5:** Means and standard deviations of PedsQL GCM total score and scale scores (*n* = 101).

	Physical functioning	Emotional functioning	Social functioning	School functioning	Total score
**Paternal educational level**					
Low (*N* = 14)	47.99 (22.25)	43.21 (17.61)	32.14 (20.35)	26.19 (25.36)	39.74 (16.13)
Middle (*N* = 16)	62.11 (21.95)	54.38 (24.96)	40.31 (22.47)	34.48 (26.43)	50.90 (17.96)
High (*N* = 71)	61.27 (19.63)	61.41 (20.65)	41.34 (22.68)	39.44 (26.48)	52.92 (15.71)
*p*-value	0.077	0.012	0.374	0.216	0.023
**Maternal educational level**					
Low (*N* = 13)	45.43 (17.80)	47.31 (15.49)	43.85 (21.62)	30.00 (25.88)	43.02 (14.61)
Middle (*N* = 14)	68.53 (19.63)	64.29 (25.10)	41.07 (22.72)	38.69 (26.11)	55.77 (17.79)
High (*N* = 74)	60.35 (20.35)	58.38 (21.65)	38.99 (22.62)	37.66 (26.81)	51.18 (16.43)
*p*-value	0.011	0.115	0.757	0.609	0.125
**Having siblings**					
No (N = 53)	59.91 (16.48)	53.68 (19.98)	37.92 (23.52)	35.72 (27.01)	49.45 (14.29)
Yes (N = 48)	59.18 (24.70)	62.29 (22.92)	42.08 (21.01)	38.02 (26.10)	52.23 (18.86)
*p*-value	0.861	0.046	0.353	0.665	0.402
**Annual household income (US dollars)**					
Less than 14,500 (*n* = 32)	53.03 (21.89)	51.09 (22.49)	37.50 (23.31)	32.92 (26.49)	45.69 (16.10)
14,500 to 36,300 (*n* = 37)	61.23 (16.38)	56.62 (20.58)	36.76 (21.06)	34.32 (25.25)	49.86 (15.07)
36,300 to 72,700 (*n* = 21)	60.12 (23.65)	64.29 (20.20)	46.67 (23.47)	44.29 (29.71)	55.43 (19.77)
More than 72,700 (*n* = 11)	71.88 (19.91)	68.64 (21.34)	44.55 (20.91)	42.27 (23.49)	59.72 (11.48)
*p*-value	0.059	0.049	0.324	0.372	0.044
**Perceived financial burden**					
Easily affordable (*N* = 8)	76.56 (17.76)	68.75 (18.47)	47.50 (18.13)	47.29 (25.38)	62.99 (9.34)
Affordable (*N* = 59)	62.34 (19.69)	60.76 (20.02)	42.97 (21.56)	36.69 (27.41)	53.20 (16.37)
Hard to afford (*N* = 34)	50.74 (19.61)	50.00 (23.45)	32.79 (23.33)	34.56 (25.19)	43.69 (15.70)
*p*-value	0.001	0.022	0.063	0.477	0.002
**School type**					
Normal school (*N* = 51)	61.76 (18.08)	60.29 (20.33)	40.78 (22.81)	47.16 (20.55)	53.72 (15.57)
Special school/rehabilitation school (*N* = 24)	60.81 (23.35)	56.88 (22.98)	33.96 (24.58)	30.97 (26.61)	48.73 (18.76)
Not going to school yet (*N* = 13)	58.17 (19.20)	60.77 (21.10)	45.77 (14.12)	19.23 (26.22)	50.27 (13.45)
Others (*N* = 13)	50.00 (25.83)	46.54 (24.27)	41.54 (22.86)	24.62 (31.90)	43.47 (18.03)
*p*-value	0.324	0.219	0.438	<0.001	0.213
**Receiving social benefit/bonuses/subsidies**					
No (*N* = 75)	59.96 (20.59)	58.67 (20.47)	44.20 (20.65)	37.44 (27.43)	52.27 (16.27)
Yes (*N* = 26)	58.41 (21.34)	55.19 (25.36)	27.50 (22.81)	35.00 (23.92)	46.46 (17.07)
*p*-value	0.745	0.486	0.001*	0.687	0.125
**Having symptoms of attention deficit and/or hyperactivity**					
No (*N* = 59)	59.32 (19.36)	57.97 (22.67)	43.90 (21.01)	34.15 (28.16)	51.26 (16.77)
Yes (*N* = 42)	59.90 (22.66)	57.50 (20.67)	34.29 (23.21)	40.56 (23.74)	50.09 (16.51)
*p*-value	0.892	0.916	0.032	0.233	0.729
**Sleeping problems**					
No (*N* = 63)	63.49 (18.38)	62.46 (19.79)	41.59 (22.18)	41.96 (23.44)	54.41 (14.38)
Yes (*N* = 38)	53.04 (22.82)	50.00 (22.87)	37.11 (22.65)	28.29 (29.23)	44.73 (18.38)
*p*-value	0.013	0.005	0.331	0.011	0.004

* Values presented in this table are means and standard deviations. Regarding the educational level, low means no education, primary school or middle school; middle level means high school, technical school, vocational secondary school and vocational high school; high level means bachelor degree or above according to the Chinese Standard Classification of Education.

Table 6 shows the associations of selected variables with the score of PedsQL GCM and FIM in the multivariate linear regression models. Having sleeping problems was statistically significantly associated with the total score of PedsQL 4.0 GCM (β:−8.43; 95% CI: −14.83, −2.03; *p* = 0.010). Children’s gender, paternal education, number of family members in the same household, annual household income, and having diseases at birth were not statistically significantly associated with the total score of PedsQL 4.0 GCM (*p*-values > 0.05). Perceived financial burden was a statistically significant associated factor of the total score of PedsQL FIM, more specifically, comparing with those who could easily afford the disease-related cost, those who could hardly afford it had significantly lower score of FIM (β:−29.16; 95% CI: −43.79, −14.53; *p* < 0.001); and those who thought the cost affordable had relatively low score of FIM (β:−13.67; 95% CI: −27.25, −0.09; *p* = 0.049). Paternal education, annual household income and perceived financial burden were not statistically significantly associated with the total score of PedsQL GCM (*p*-values > 0.05).

**Table 6:** Associations of selected variables with the total score of PedsQL GCM and FIM by multivariate linear regression analyses.

	Total score of PedsQL FIM		Total score of PedsQL GCM	
	β (95% CI)	*p-*value	β (95% CI)	*p*-value
**Gender**				
Boy (*n* = 66)	Reference		\	\
Girl (*n* = 35)	6.19 (−1.04, 13.42)	0.092	\	\
**Paternal educational level**				
High level (*n* = 71)	Reference		Reference	
Mid level (*n* = 16)	−3.22 (−12.73, 6.28)	0.502	−1.21 (−9.95, 7.53)	0.784
Low level (*n* = 14)	−9.04 (−20.04, 1.95)	0.106	−9.45 (−19.33, 0.44)	0.061
**Numbers of family members living in the same household**				
Three or less (*n* = 19)	Reference		\	\
Four (*n* = 35)	5.77 (−3.89, 15.42)	0.239	\	\
Five (*n* = 25)	4.31 (−6.06, 14.69)	0.411	\	\
Six and more (*n* = 22)	9.30 (−1.64, 20.23)	0.095	\	\
**Annual household income (US dollars)**				
More than 72,700 (*n* = 11)	Reference		Reference	
36,300 to 72,700 (*n* = 21)	−0.28 (−12.84, 12.28)	0.965	−0.11 (−11.67, 11.46)	0.985
14,500 to 36,300 (*n* = 37)	−2.23 (−14.21, 9.74)	0.712	−4.19 (−15.09, 6.70)	0.447
Less than 14,500 (*n* = 32)	−11.14 (−24.94, 2.66)	0.112	−3.20 (−15.76, 9.36)	0.614
**Perceived financial burden**				
Easily affordable (*n* = 8)	Reference		Reference	
Affordable (*n* = 59)	−13.67 (−27.25, −0.09)	0.049	−5.77 (−17.53, 6.00)	0.333
Hard to afford (*n* = 34)	−29.16 (−43.79, −14.53)	<0.001	−12.71 (−25.97, 0.55)	0.06
**Having diseases at birth**				
No (*n* = 23)	Reference		\	\
Yes (*n* = 78)	4.71 (−3.52, 12.94)	0.259	\	\
**Sleeping problems**				
No (*N* = 63)	\	\	Reference	
Yes (*N* = 38)	\	\	−8.43 (−14.83, −2.03)	0.010
**Adjusted R**^**2**^	0.436		0.161	

CI, confident interval.

## 4. Discussion

The present study assessed proxy-reported HRQoL of children with Williams syndrome and self-reported HRQoL of caregivers in a relatively large sample of Chinese pediatric patients. To our best knowledge, it was the first study to assess HRQoL in children with WS. In addition, this study demonstrated the associated factors of HRQoL of children with WS and their caregivers. To be noted, having sleeping problems was significantly associated with poor HRQoL of children, and when caregivers perceived the medical cost as hardly unaffordable, their self-reported HRQoL was remarkably worse than those who could easily afford for the cost.

We found that the HRQoL of children with WS and their caregivers were significantly worse than the healthy population as a reference regarding each domain and the overall HRQoL. As indicated by effect sizes, differences in caregiver’s HRQoL can be interpreted as large differences in terms of its clinical relevance in all scales and total scores of PedsQL 3.0 FIM except for the scales of cognitive functioning and family relationship that can be interpreted as moderate differences. Differences in proxy-reported HRQoL of children with WS can be interpreted as large differences in our study. Notably, WS can profoundly impact the physical, psychological, social, and role functioning of both children and their caregivers. Due to the nature of this disease, WS can cause impairments in the biological structure and functioning of multiple systems, such as cardiovascular and endocrine systems, which may lead to a poor prognosis. If there were severe damages to organs, in particular, cardiovascular damages, children may have high morbidity and mortality and often die of heart failure. Thus, raising and caring for a child with WS imposes heavy distress and financial burden, and required more involvement of parents’ personal time which together may impair the family functioning.

### 4.1. Health-related quality of life of caregivers of children with WS

Our study showed that the HRQoL of caregivers of male patients was worse than that of female patients regarding physical, emotional, and social functioning, worry domains, and overall HRQoL. Although WS occurs equally in both genders, raising a boy with WS seems more challenging than raising a girl. This finding might be explained by the gender differences in children’s growth and behaviors as well as the role expectations by society. Boys are generally naughtier and more active, and in Chinese social norms, girls are encouraged to be sensible and disciplined. Therefore, raising a boy with WS may cause more distress to caregivers’ physical, emotional, and social health and overall HRQoL than raising a girl.

We found the average scores of physical, social, cognitive functioning, communication, and family relationship scales and total score were significantly lower (*p*-values < 0.05) when the father had a low educational level (i.e., no primary education school and middle school) compared with fathers with high education (i.e., bachelor degree or above); the effect sizes range from 0.48 to 1.10 indicating a moderate to large differences. While regarding maternal educational levels, the statistically significant difference was only observed in the score of the cognitive functioning scale (*p* < 0.05). Our finding was consistent with the Brazilian study showing paternal education positively related to family quality of life (10).

We did not find a significant difference in the caregiver’s HRQoL across marital status (i.e., being married vs. being divorced or widowed). However, results in our study showed that the scores of physical and cognitive functioning scales were higher in parents always living together than those only sometimes living together (*p*-values < 0.05), and effect sizes were 0.81 and 0.93 (Supplementary Table S2), which indicated a large difference. This finding was interesting and might be related to Chinese culture. There have been some couples who got divorced but still lived together for the child’s good. Another explanation was that the small sample size of the subgroup of parents who were divorced or widowed might lead to a chance finding.

Our study highlighted significant disparities in caregivers’ HRQoL across families with different levels of financial burden as indicated by the annual household income, perceived financial burden due to the disease, and the residence (urban or rural) areas. Especially the effect sizes ranged from 1.12 to 2.43 (see Supplementary Table S2), which indicated large differences when comparing caregivers’ HRQoL domain and total scores between the families that can easily afford and those hardly afford. Large differences were observed in all domain scores of caregivers’ HRQoL and the overall score between the highest and the lowest subgroup of household income (i.e., more than 72,700 vs. less than 14,500 US dollars per year), with the effect sizes ranging from 1.03 to 2.02. The above finding was consistent with the Brazilian study reporting family income as a determinant of the family’s quality of life with WS children (10). They also found that supplemental health insurance and social benefits were associated with better family quality of life, which was not confirmed by our study. It may be explained that China and Brazil have different health insurance systems and policies regarding rare diseases.

Regarding the disease-related characteristics, our study showed that having diseases at birth was related to poorer physical functioning, emotional functioning, and communication within the family, and these differences were interpreted as moderate, with effect sizes ranging from 0.58 to 0.66. Diseases at birth included preterm birth, small for gestational age (SGA), neonatal jaundice, asphyxia, hypothyroidism, congenital heart disease, and indirect inguinal hernia. As mentioned above, children with one or more disorders may have a higher demand for medical care and family care, which may burden the caregiver’s physical and emotional health and influence communication among the family members. Children with sleep problems were related to a worse family relationship than those without such an issue; the difference was small according to the effect size (*d* = 0.49). According to a systematic review by Coles et al., poor sleeping quality of children was negatively associated with the quality of family relationships (i.e., between parent and child and within the couple) (18).

Results from the multivariate linear regression analysis showed that the perceived financial burden due to the treatment of WS was significantly independently associated with self-reported HRQoL of caregivers after adjusting other selected variables. This indicated the remarkable influence of perceived financial burden on caregivers’ and family’s quality of life regardless of the other objective indicators of socioeconomic status (e.g., education and household income) and disease-related characteristics. By now, there was no such financial support feasible in China. Regarding other rare diseases or certain disorders, for instance, autism, the Federation of Disabled Persons and foundations provide certain amount of subsidies for families with sick children to support for medical care and rehabilitation. Therefore, social support, in particular, social benefits, subsidies and health insurances that may cover some costs of WS treatment and care, is warranted.

### 4.2. Health-related quality of life of children with WS

To the best knowledge, the present study was the first to measure the HRQoL of children with WS. It provided comprehensive data on multiple domains (i.e., physical, emotional, social, and school functioning) of children’s HRQoL. Socio-economic status as indicated by education and income was an important factor. Our study showed that the emotional functioning and overall HRQoL of children were poorer in children whose fathers had low educational level than those whose father had high education, and the differences were large (*p*-values < 0.05; effect sizes: 0.88 and 0.82, see Supplementary Tables S4, S5). The subjective indicator (i.e., perceived financial burden) seemed more sensitive than the objective measurement (i.e., household income) in distinguishing the subgroup differences in children’s HRQoL. More specifically, the *post hoc* test did not find significant differences in PedsQL 4.0 GCM scale and total scores between every two subgroups regarding the annual household income (*p*-values > 0.05, data not shown). We found that children’s physical functioning and overall HRQoL were significantly higher in the family who easily afforded the cost of taking care of WS children than those who hardly afforded (*p*-value < 0.001, effect size = 1.32; *p*-value < 0.05; effect size = 1.23; see Supplementary Tables S4, S5). Our results suggested that the perception of financial burden related to caring WS children may be a more sensitive indicator of children’s HRQoL than the objective measurement such as household income. Having attention deficit and/or hyperactivity was related to the impaired social functioning of children, but the effect size indicated small difference. Sleeping problems may decrease children’s overall HRQoL, specifically physical, emotional and school functioning, while the differences were small or moderate. To be noted, the presence of sleeping problems was the only statistically significant factor in the multivariate regression model, which indicated the very important impact of sleeping on WS children’s HRQoL. This finding also inspired us to develop targeted intervention to improve quality of life of these children by improving the sleeping quality.

### 4.3. Strengths and limitations

Our study has several strengths. The present study was one of the few to assess the HRQoL of children with WS and the family impact by the disease using reliable and valid QoL measurements (i.e., PedsQL 3.0 FIM and PedsQL 4.0 GCM). The sample size was relatively large, with 101 child-caregiver dyads, which, to our knowledge, was the largest sample regarding HRQoL of children with WS. Our study provided affluent data of domain scores and total scores of HRQoL of both caregivers and children, and we compared our data with healthy control at the same age. In addition, a comprehensive set of variables regarding social demographic and clinical characteristics were included in the analyses, which was a preliminary attempt to identify the associated factors with HRQoL. *Post hoc* analyses were adopted to identify the difference between the exact two groups, and effect size (Cohen’s *d*) was used to evaluate the clinical relevance.

Several limitations should be taken into account. First, most caregivers participating in this study had a high educational level (i.e., bachelor’s degree or higher), which may not represent the population of children with WS and their families. This may be explained by the fact that caregivers with relatively high education may have more resources and higher health literacy and are more likely to seek medical care. Selection bias may occur, so the interpretation of our results should be made with caution. Thirdly, causation cannot be concluded due to the cross-sectional study design.

To summarize, our study has comprehensively assessed the HRQoL of Chinese children with WS and of their caregivers as well as the potential determinants of HRQoL. We highlighted that the HRQoL of children with WS and their caregivers was dramatically worse than the healthy references. Paternal educational level, household income, and the perceived financial burden significantly influence the HRQoL of both children and families. Therefore, we call for attention from policymakers and other stakeholders on the health status and well-being of children with WS and their families. Supports are needed to relieve psychosocial distress and financial burden.

## Data availability statement

The raw data supporting the conclusions of this article will be made available by the authors, without undue reservation.

## Ethics statement

The studies involving human participants were reviewed and approved by the Ethics Committee of the Children’s Hospital of Zhejiang University School of Medicine (2019-IRB-122). Written informed consent to participate in this study was provided by the participants’ legal guardian/next of kin.

## Author contributions

WC, CZ, and GB were involved in the study conceptualization and study design. WC, CJ, FL, and JS collected data. XH, ZL, and YD carried out statistical analyses. WC, LS, and GB wrote the first draft of the article. WC, LS, XH, TP, XJ, LH, CZ, and GB provided critical revisions of the article for important intellectual content. All authors contributed to the article and approved the submitted version.

## Funding

GB received the starting grant for research from the Children’s Hospital, Zhejiang University School of Medicine, National Children’s Regional Medical Center, and National Clinical Research Center for Child Health. The funders had no role in the study design; data collection, analysis, or interpretation; in the writing of the report; or in the decision to submit the article for publication.

## Conflict of interest

The authors declare that the research was conducted in the absence of any commercial or financial relationships that could be construed as a potential conflict of interest.

## Publisher’s note

All claims expressed in this article are solely those of the authors and do not necessarily represent those of their affiliated organizations, or those of the publisher, the editors and the reviewers. Any product that may be evaluated in this article, or claim that may be made by its manufacturer, is not guaranteed or endorsed by the publisher.
