# Supplementary material for: Health-related quality of life of children with Williams syndrome and caregivers in China
Source: Front Public Health. 2023 Jun 9;11:1177317. doi: 10.3389/fpubh.2023.1177317 (PMC10288101; doi:10.3389/fpubh.2023.1177317)
Supplement: Supplementary file 1 [file Table_1.DOCX]

**Legends of supplementary tables**

Supplementary Table S1. Pairwise difference in PedsQL 3.0 FIM scales and total score (n=101)

Supplementary Table S2. Effect sizes for subgroup differences of PedsQL 3.0 FIM scales and total score (n=101)

Supplementary Table S3. Means and standard deviations of PedsQL 3.0 FIM total scores and domain scores (n=101) for non-significant variables

Supplementary Table S4. Pairwise difference in PedsQL 4.0 GCM scales and total score (n=101)

Supplementary Table S5. Effect sizes for subgroup differences of PedsQL 4.0 GCM scales and total scores (n=101)

Supplementary Table S6. Means and standard deviations of PedsQL 4.0 GCM total scores and scale scores for non-significant variables (n=101)

**Supplementary Table S1. Pairwise difference in PedsQL 3.0 FIM scales and total score (n=101)**

|  | Physical functioning | Emotional functioning | Social functioning | Cognitive functioning | Communication | Worry | Daily activities | Family relationships | Total score |
| --- | --- | --- | --- | --- | --- | --- | --- | --- | --- |
| Gender  14.5**  11.11*  11.96*  9.68  14.84*  12.6*  8.91  7 | | | | | | | | | |
| Girl vs Boy | 14.50** | 11.11* | 11.96* | 9.68 | 14.84* | 12.60* | 8.91 | 7.52 | 11.39* |
| Paternal educational level |  |  |  |  |  |  |  |  |  |
| Middle vs Low | 16.41 | 1.70 | 13.78 | 21.16* | 12.13 | 7.05 | 15.77 | 18.44 | 13.30 |
| High vs Low | 23.39** | 12.76 | 19.69* | 28.19*** | 25.13* | 14.84 | 14.92 | 22.18* | 20.14*** |
| High vs Middle | 6.98 | 11.07 | 5.90 | 7.03 | 13.01 | 7.79 | -0.85 | 3.75 | 6.83 |
| Maternal educational level |  |  |  |  |  |  |  |  |  |
| Middle vs Low | 2.11 | -4.42 | -3.06 | 2.23 | 2.29 | -4.42 | 7.42 | 17.01 | 2.39 |
| High vs Low | 11.81 | 9.36 | 9.75 | 16.69 | 11.56 | 1.79 | 8.58 | 12.60 | 10.27 |
| High vs Middle | 9.70 | 13.78 | 12.80 | 14.47 | 9.27 | 6.22 | 1.16 | -4.40 | 7.87 |
| Living together |  |  |  |  |  |  |  |  |  |
| Sometimes vs Always | -21.03* | -14.39 | -7.21 | -23.35* | -17.52 | -15.6 | -9.50 | -12.63 | -15.15 |
| Never vs Always | 12.72 | -7.39 | 12.79 | -3.85 | 2.90 | -10.85 | -2.42 | -21.88 | -2.25 |
| Never vs Sometimes | 33.75 | 7.00 | 20.00 | 19.50 | 20.42 | 4.75 | 7.08 | -9.25 | 12.91 |
| Numbers of family members living in the same household | | | | | | | | | |
| Four vs Three or less | 9.29 | 14.29 | 6.81 | 19.95* | 10.51 | 7.26 | 14.49 | 18.28* | 12.61 |
| Five vs Three or less | -2.35 | 12.18 | 2.17 | 3.53 | 7.23 | 6.06 | 10.77 | 8.62 | 6.03 |
| Six and more vs Three or less | 14.38 | 31.12*** | 11.60 | 18.94 | 19.26 | 17.76 | 20.89* | 15.92 | 18.74* |
| Five vs Four | -11.64 | -2.11 | -4.64 | -16.43 | -3.29 | -1.20 | -3.71 | -9.66 | -6.59 |
| Six and more vs Four | 5.09 | 16.83 | 4.79 | -1.02 | 8.74 | 10.50 | 6.41 | -2.36 | 6.12 |
| Six and more vs Five | 16.73 | 18.95* | 9.43 | 15.41 | 12.03 | 11.70 | 10.12 | 7.30 | 12.71 |
| Annual household income ( US dollars) | | | | | | | | | |
| 14,500 to 36,300 vs Less than 14,500 | 19.63*** | 28.01*** | 19.85*** | 23.90*** | 25.99*** | 16.74*** | 11.11 | 17.17* | 20.30*** |
| 36,300 to 72,700 vs 14,500 to 36,300 | 29.82*** | 29.82*** | 35.43*** | 25.45*** | 31.31*** | 25.92*** | 26.71*** | 22.76*** | 28.40*** |
| More than 72,700 vs 14,500 to 36,300 | 35.11*** | 27.16* | 27.82*** | 38.69*** | 43.25*** | 27.33* | 24.76* | 32.91*** | 32.13*** |
| 36,300 to 72,700 vs 14,500 to 36,300 | 10.19 | 1.81 | 15.57 | 1.54 | 5.32 | 9.18 | 15.60 | 5.59 | 8.10 |
| More than 72,700 vs 14,500 to 36,300 | 15.48 | -0.85 | 7.97 | 14.79 | 17.26 | 10.59 | 13.66 | 15.74 | 11.83 |
| More than 72,700 vs 36,300 to 72,700 | 5.28 | -2.66 | -7.60 | 13.25 | 11.94 | 1.41 | -1.95 | 10.15 | 3.73 |
| Residence |  |  |  |  |  |  |  |  |  |
| Town or rural vs urban | -11.19* | 0.40 | -12.09* | -11.52* | -9.21 | -9.04 | -5.30 | -4.50 | -7.81 |
| Perceived financial burden |  |  |  |  |  |  |  |  |  |
| Affordable vs Easily Affordable | -20.22* | -12.28 | -15.66 | -22.71* | -16.77 | -11.93 | -18.52 | -9.63 | -15.96* |
| Hard to afford vs Easily Affordable | -47.61*** | -37.98*** | -41.59*** | -45.59*** | -46.2*** | -34.63*** | -41.12*** | -30.4*** | -40.64*** |
| Hard to afford vs Affordable | -27.39*** | -25.70*** | -25.93*** | -22.88*** | -29.43*** | -22.70*** | -22.59*** | -20.78*** | -24.67*** |
| Diseases at birth |  |  |  |  |  |  |  |  |  |
| Yes vs No | 15.15* | 17.05** | 9.21 | 9.40 | 16.79* | 9.87 | 9.18 | -0.52 | 10.77* |
| Sleeping problems |  |  |  |  |  |  |  |  |  |
| Yes vs No | -8.90 | -5.50 | -4.74 | -3.07 | -9.81 | -7.37 | -9.42 | -13.23* | -7.76 |

a: Values presented in this table is difference between two groups.

b: p<0.05 is displayed as *, p<0.01 is displayed as **, p<0.001 is displayed as ***.

c: For Paternal and Maternal educational level, Low means Middle school or lower, Middle means High school educated, High means college and higher.

**Supplementary Table S2. Effect sizes for subgroup differences of PedsQL 3.0 FIM scales and total score (n=101)**

|  | Physical functioning | Emotional functioning | Social functioning | Cognitive functioning | Communication | Worry | Daily activities | Family relationships | Total score |
| --- | --- | --- | --- | --- | --- | --- | --- | --- | --- |
| Gender |  |  |  |  |  |  |  |  |  |
| Boy vs. girl | 0.57 | 0.41 | 0.46 | 0.73 | 0.51 | 0.47 | 0.35 | 0.27 | 0.53 |
| Paternal educational level |  |  |  |  |  |  |  |  |  |
| Low vs. middle | 0.67 | 0.07 | 0.52 | 0.99 | 0.42 | 0.28 | 0.61 | 0.75 | 0.69 |
| Low vs. high | 0.89 | 0.48 | 0.74 | 1.10 | 0.89 | 0.54 | 0.60 | 0.87 | 0.93 |
| Middle vs. high | 0.27 | 0.41 | 0.23 | 0.28 | 0.45 | 0.29 | 0.03 | 0.15 | 0.32 |
| Maternal educational level |  |  |  |  |  |  |  |  |  |
| Low vs. middle | 0.07 | 0.17 | 0.12 | 0.09 | 0.08 | 0.16 | 0.29 | 0.64 | 0.11 |
| Low vs. high | 0.45 | 0.35 | 0.36 | 0.64 | 0.39 | 0.06 | 0.33 | 0.48 | 0.46 |
| Middle vs. high | 0.32 | 0.52 | 0.47 | 0.56 | 0.32 | 0.23 | 0.04 | 0.17 | 0.35 |
| Living together |  |  |  |  |  |  |  |  |  |
| Always vs. sometimes | 0.81 | 0.54 | 0.27 | 0.93 | 0.59 | 0.58 | 0.38 | 0.43 | 0.69 |
| Always vs. never | 0.38 | 0.28 | 0.34 | 0.12 | 0.09 | 0.40 | 0.06 | 0.64 | 0.09 |
| Sometimes vs. never | 1.02 | 0.32 | 0.53 | 0.62 | 0.65 | 0.21 | 0.17 | 0.27 | 0.50 |
| Number of family members living in the same household | | | | | | | | | |
| Three or less vs. four | 0.36 | 0.61 | 0.28 | 0.86 | 0.38 | 0.27 | 0.61 | 0.74 | 0.64 |
| Three or less vs. five | 0.08 | 0.39 | 0.07 | 0.13 | 0.25 | 0.24 | 0.38 | 0.29 | 0.24 |
| Three or less vs. six and more | 0.55 | 1.33 | 0.43 | 0.78 | 0.62 | 0.67 | 0.91 | 0.64 | 0.90 |
| Four vs. five | 0.41 | 0.07 | 0.15 | 0.61 | 0.11 | 0.05 | 0.13 | 0.33 | 0.26 |
| Four vs. six and more | 0.20 | 0.78 | 0.18 | 0.04 | 0.28 | 0.39 | 0.27 | 0.10 | 0.29 |
| Five vs. six and more | 0.59 | 0.60 | 0.30 | 0.57 | 0.39 | 0.44 | 0.35 | 0.25 | 0.50 |
| Annual household income (US dollars) | | | | | | | | | |
| Less than 14,500 vs. 14,500 to 36,300 | 0.83 | 1.13 | 0.84 | 1.08 | 0.91 | 0.62 | 0.46 | 0.66 | 1.05 |
| Less than 14,500 vs. 36,300 to 72,700 | 1.30 | 1.30 | 1.50 | 1.02 | 1.09 | 1.02 | 1.08 | 0.88 | 1.38 |
| Less than 14,500 vs. more than 72,700 | 1.53 | 1.05 | 1.04 | 2.02 | 1.92 | 0.93 | 1.03 | 1.27 | 1.75 |
| 14,500 to 36,300 vs. 36,300 to 72,700 | 0.43 | 0.07 | 0.69 | 0.07 | 0.19 | 0.34 | 0.58 | 0.25 | 0.39 |
| 14,500 to 36,300 vs. more than 72,700 | 0.66 | 0.03 | 0.30 | 0.69 | 0.60 | 0.36 | 0.58 | 0.71 | 0.61 |
| 36,300 to 72,700 vs. more than 72,700 | 0.23 | 0.10 | 0.28 | 0.53 | 0.48 | 0.05 | 0.08 | 0.47 | 0.18 |
| Residence |  |  |  |  |  |  |  |  |  |
| Town or rural vs. urban | 0.42 | 0.01 | 0.47 | 0.44 | 0.32 | 0.33 | 0.20 | 0.17 | 0.35 |
| Perceived financial burden |  |  |  |  |  |  |  |  |  |
| Easily affordable vs. affordable | 0.92 | 0.52 | 0.70 | 1.04 | 0.64 | 0.46 | 0.84 | 0.45 | 0.88 |
| Easily affordable vs. hard to afford | 2.21 | 1.73 | 1.77 | 1.98 | 2.00 | 1.39 | 1.89 | 1.12 | 2.43 |
| Affordable vs. hard to afford | 1.25 | 1.08 | 1.10 | 0.99 | 1.13 | 0.88 | 1.02 | 0.77 | 1.36 |
| Diseases at birth |  |  |  |  |  |  |  |  |  |
| No vs. yes | 0.58 | 0.66 | 0.32 | 0.37 | 0.58 | 0.36 | 0.36 | 0.02 | 0.49 |
| Sleeping problems |  |  |  |  |  |  |  |  |  |
| No vs. yes | 0.34 | 0.21 | 0.17 | 0.12 | 0.34 | 0.28 | 0.37 | 0.49 | 0.34 |

**Supplementary Table S3. Means and standard deviations of PedsQL 3.0 FIM total scores and domain scores (n=101) for non-significant variables**

|  | Physical functioning | Emotional functioning | Social functioning | Cognitive functioning | Communication | Worry | Daily activities | Family relationships | | Total score |
| --- | --- | --- | --- | --- | --- | --- | --- | --- | --- | --- |
| Parental marital status | | | | | | | | | | |
| Married (N=96) | 58.55 (25.89) | 48.59 (26.45) | 55.99 (26.28) | 61.67 (25.54) | 51.56 (29.13) | 37.81 (26.55) | 54.25 (24.99) | 64.53 (24.91) | 54.12 (21.93) | |
| Divorced or Widowed (N=5) | 60.00 (33.67) | 40.00 (18.37) | 51.25 (29.45) | 56.00 (27.25) | 55.00 (27.39) | 34.00 (21.62) | 38.33 (25.41) | 44.00 (28.81) | 47.32 (21.77) | |
| *p* value | 0.904 | 0.476 | 0.697 | 0.631 | 0.797 | 0.753 | 0.168 | 0.077 | 0.501 | |
| Having siblings |  |  |  |  |  |  |  |  |  | |
| No (N=53) | 57.55 (23.98) | 43.96 (22.90) | 55.07 (25.65) | 57.92 (24.48) | 48.43 (26.10) | 33.40 (22.44) | 51.57 (24.63) | 60.66 (25.00) | 51.07 (19.91) | |
| Yes (N=48) | 59.81 (28.51) | 52.81 (28.79) | 56.51 (27.26) | 65.21 (26.34) | 55.38 (31.63) | 42.29 (29.44) | 55.56 (25.75) | 66.67 (25.63) | 56.78 (23.69) | |
| *p* value | 0.666 | 0.089 | 0.785 | 0.153 | 0.229 | 0.089 | 0.429 | 0.236 | 0.192 | |
| Health Insurance | | | | | | | | | | |
| No (N=21) | 57.94 (23.27) | 50.95 (26.44) | 54.76 (25.46) | 62.86 (22.78) | 52.38 (29.60) | 38.33 (26.89) | 51.19 (25.18) | 60.24 (24.82) | 53.58 (20.60) | |
| Yes (N=80) | 58.80 (26.95) | 47.44 (26.14) | 56.02 (26.67) | 61.00 (26.31) | 51.56 (28.93) | 37.44 (26.25) | 54.06 (25.23) | 64.38 (25.57) | 53.84 (22.31) | |
| *p* value | 0.893 | 0.586 | 0.847 | 0.768 | 0.909 | 0.890 | 0.643 | 0.508 | 0.962 | |
| Receiving social benefits/bonuses/subsidies | | | | | | | | | | |
| No (N=75) | 58.44 (26.80) | 47.20 (25.64) | 56.50 (26.31) | 60.13 (25.44) | 50.78 (26.54) | 36.07 (25.37) | 54.33 (25.13) | 62.20 (26.04) | 53.21 (21.72) | |
| Yes (N=26) | 59.13 (24.55) | 50.96 (27.75) | 53.61 (26.70) | 65.00 (25.88) | 54.49 (35.37) | 42.12 (28.71) | 50.96 (25.42) | 67.31 (23.33) | 55.45 (22.65) | |
| *p* value | 0.908 | 0.529 | 0.631 | 0.405 | 0.576 | 0.314 | 0.558 | 0.379 | 0.655 | |
| Cost related to WS treatment per year (US dollars) | | | | | | | | | | |
| Less than 14,500 (N=91) | 60.12 (25.95) | 49.12 (25.69) | 56.39 (25.92) | 61.59 (26.17) | 53.57 (28.68) | 38.41 (26.06) | 53.48 (25.37) | 63.02 (26.03) | 54.46 (21.82) | |
| 14,500 to 32,300 (N=10) | 45.00 (24.91) | 39.50 (29.76) | 50.00 (30.48) | 59.50 (19.50) | 35.00 (26.87) | 30.50 (28.33) | 53.33 (23.96) | 68.00 (18.44) | 47.60 (22.46) | |
| *p* value | 0.082 | 0.271 | 0.469 | 0.807 | 0.053 | 0.369 | 0.986 | 0.558 | 0.349 | |
| School type | | | | | | | | | | |
| Normal school (N=51) | 62.50 (24.25) | 47.94 (25.08) | 54.17 (23.94) | 62.16 (26.58) | 50.16 (29.32) | 37.75 (25.62) | 51.63 (22.24) | 64.51 (23.84) | 53.85 (20.07) | |
| Special Education School or Recovery Education School (N=24) | 54.69 (26.84) | 47.92 (26.94) | 58.59 (27.26) | 61.88 (25.01) | 55.90 (31.23) | 38.12 (29.67) | 54.86 (26.11) | 62.50 (26.95) | 54.31 (23.55) | |
| Not go to school yet (N=13) | 61.86 (25.51) | 57.31 (26.19) | 65.38 (25.72) | 63.85 (23.64) | 60.26 (27.25) | 45.00 (23.09) | 67.31 (26.89) | 69.62 (25.29) | 61.32 (23.25) | |
| Others (N=13) | 47.44 (31.02) | 40.38 (28.76) | 47.12 (32.94) | 55.00 (25.90) | 41.67 (23.32) | 28.85 (25.43) | 44.23 (29.14) | 55.38 (28.97) | 45.01 (23.62) | |
| *p* value | 0.237 | 0.436 | 0.310 | 0.808 | 0.344 | 0.483 | 0.108 | 0.537 | 0.305 | |
| Having a genetic test | | | | | | | | | | |
| No (N=10) | 58.33 (18.00) | 52.00 (15.13) | 56.25 (24.12) | 62.00 (19.18) | 48.33 (31.13) | 35.00 (18.26) | 50.00 (22.57) | 53.00 (24.74) | 51.86 (16.57) | |
| Yes (N=91) | 58.65 (26.94) | 47.75 (27.07) | 55.70 (26.66) | 61.32 (26.20) | 52.11 (28.83) | 37.91 (27.05) | 53.85 (25.47) | 64.67 (25.29) | 53.99 (22.44) | |
| *p* value | 0.971 | 0.627 | 0.950 | 0.937 | 0.697 | 0.741 | 0.648 | 0.168 | 0.772 | |
| Getting rehabilitation | | | | | | | | | | |
| No (N=41) | 59.65 (24.94) | 46.10 (24.17) | 55.18 (26.81) | 61.10 (23.55) | 50.00 (27.89) | 35.24 (23.34) | 53.66 (23.87) | 62.07 (24.47) | 52.88 (19.85) | |
| Yes (N=60) | 57.92 (27.09) | 49.58 (27.47) | 56.15 (26.18) | 61.58 (26.97) | 52.92 (29.78) | 39.25 (28.15) | 53.33 (26.14) | 64.50 (26.10) | 54.40 (23.29) | |
| *p* value | 0.744 | 0.513 | 0.858 | 0.926 | 0.621 | 0.454 | 0.949 | 0.639 | 0.732 | |
| Congenital heart disease and surgery | | | | | | | | | | |
| No (N=18) | 54.63 (25.56) | 45.28 (25.29) | 53.12 (28.22) | 65.83 (24.03) | 53.70 (22.91) | 38.89 (26.65) | 49.54 (26.11) | 69.44 (22.74) | 53.80 (20.45) | |
| Having CHD but no surgery (N=63) | 61.57 (26.55) | 50.56 (26.94) | 56.94 (27.01) | 62.06 (26.30) | 51.85 (30.73) | 36.90 (27.29) | 54.10 (25.74) | 62.54 (26.15) | 54.57 (22.87) | |
| Having CHD and surgery, receiving (N=20) | 52.92 (25.08) | 43.25 (24.40) | 54.38 (23.13) | 55.25 (24.36) | 49.58 (29.05) | 38.75 (23.61) | 55.00 (23.01) | 61.25 (25.44) | 51.30 (20.68) | |
| *p* value | 0.340 | 0.487 | 0.836 | 0.421 | 0.909 | 0.940 | 0.761 | 0.543 | 0.847 | |
| Presence of attention deficit and hyperactivity | | | | | | | | | | |
| No (N=59) | 56.00 (26.15) | 47.03 (24.51) | 56.67 (26.00) | 58.81 (24.45) | 50.14 (25.02) | 36.02 (24.12) | 53.95 (24.48) | 63.98 (24.42) | 52.83 (20.11) | |
| Yes (N=42) | 62.30 (25.95) | 49.76 (28.43) | 54.46 (26.99) | 65.00 (26.82) | 53.97 (33.85) | 39.88 (29.14) | 52.78 (26.27) | 62.86 (26.90) | 55.13 (24.31) | |
| *p* value | 0.234 | 0.607 | 0.680 | 0.232 | 0.515 | 0.469 | 0.818 | 0.827 | 0.605 | |
| Visual system conditions | | | | | | | | | | |
| Normal (N=17) | 64.46 (30.37) | 47.65 (25.62) | 57.35 (30.32) | 67.65 (30.93) | 50.49 (33.00) | 37.65 (24.37) | 57.84 (28.02) | 74.41 (21.35) | 57.19 (24.74) | |
| Abnormal (N=29) | 58.19 (24.26) | 46.03 (25.40) | 51.72 (23.97) | 60.52 (25.08) | 48.28 (31.92) | 33.79 (28.62) | 47.99 (17.49) | 59.48 (24.90) | 50.75 (20.63) | |
| Not tested (N=55) | 57.05 (25.92) | 49.45 (27.01) | 57.39 (26.44) | 59.91 (24.10) | 53.94 (26.20) | 39.64 (25.76) | 55.00 (27.43) | 62.27 (26.19) | 54.33 (21.79) | |
| *p* value | 0.594 | 0.849 | 0.625 | 0.542 | 0.686 | 0.629 | 0.353 | 0.135 | 0.610 | |
| Endocrinologic system conditions | | | | | | | | | | |
| No (N=44) | 60.51 (26.08) | 48.18 (25.20) | 56.25 (24.63) | 61.36 (25.91) | 51.33 (26.71) | 38.30 (25.03) | 56.82 (22.68) | 68.86 (20.40) | 55.20 (19.54) | |
| Yes (N=57) | 57.16 (26.29) | 48.16 (27.02) | 55.37 (27.74) | 61.40 (25.44) | 52.05 (30.75) | 37.11 (27.37) | 50.88 (26.76) | 59.39 (28.08) | 52.69 (23.62) | |
| *p* value | 0.526 | 0.996 | 0.869 | 0.994 | 0.902 | 0.823 | 0.240 | 0.062 | 0.570 | |
| Inguinal hernia | | | | | | | | | | |
| No (N=51) | 59.56 (25.13) | 46.67 (23.59) | 56.50 (25.00) | 60.49 (25.79) | 53.92 (27.86) | 37.35 (24.28) | 54.08 (22.75) | 63.53 (24.85) | 54.01 (19.42) | |
| Yes (N=28) | 59.23 (25.84) | 54.46 (24.24) | 58.04 (24.99) | 64.11 (26.53) | 54.46 (28.10) | 43.04 (26.05) | 52.98 (26.47) | 60.71 (23.32) | 55.88 (22.88) | |
| Not tested (N=22) | 55.68 (29.59) | 43.64 (32.96) | 51.14 (31.20) | 60.00 (24.45) | 43.18 (31.98) | 31.36 (30.44) | 52.65 (29.48) | 67.05 (29.47) | 50.59 (26.26) | |
| *p* value | 0.838 | 0.295 | 0.633 | 0.803 | 0.294 | 0.297 | 0.969 | 0.685 | 0.698 | |
| Repeated respiratory tract infection | | | | | | | | | | |
| No (N=77) | 60.01 (24.55) | 49.42 (25.48) | 57.22 (26.42) | 62.92 (24.12) | 53.46 (28.30) | 38.77 (26.07) | 53.14 (25.33) | 65.06 (24.57) | 55.00 (20.80) | |
| Yes (N=24) | 54.17 (30.79) | 44.17 (28.23) | 51.04 (25.91) | 56.46 (29.58) | 46.18 (30.79) | 33.96 (27.07) | 54.51 (24.94) | 58.54 (27.68) | 49.88 (25.09) | |
| *p* value | 0.341 | 0.393 | 0.317 | 0.281 | 0.284 | 0.436 | 0.816 | 0.273 | 0.319 | |
| Digestive system conditions | | | | | | | | | | |
| No (N=76) | 58.22 (25.72) | 47.89 (26.08) | 57.57 (27.01) | 61.51 (25.79) | 51.54 (28.89) | 39.08 (27.06) | 53.40 (25.50) | 64.93 (24.39) | 54.27 (21.72) | |
| Yes (N=25) | 59.83 (27.82) | 49.00 (26.73) | 50.25 (23.70) | 61.00 (25.17) | 52.33 (29.61) | 33.20 (23.58) | 53.67 (24.42) | 59.20 (28.16) | 52.31 (22.71) | |
| *p* value | 0.791 | 0.855 | 0.230 | 0.931 | 0.905 | 0.334 | 0.963 | 0.329 | 0.700 | |
| Urinary system conditions | | | | | | | | | | |
| No (N=55) | 59.02 (25.21) | 48.27 (23.12) | 60.11 (23.68) | 62.18 (24.43) | 53.94 (28.09) | 39.09 (24.13) | 56.36 (24.58) | 64.36 (22.15) | 55.42 (19.79) | |
| Yes (N=8) | 72.92 (27.73) | 58.75 (31.71) | 62.50 (24.32) | 70.00 (22.52) | 64.58 (33.26) | 52.50 (33.38) | 65.62 (16.33) | 72.50 (22.20) | 64.92 (21.87) | |
| Not tested (N=38) | 55.04 (26.71) | 45.79 (29.03) | 48.03 (28.99) | 58.42 (27.68) | 45.83 (28.66) | 32.37 (26.88) | 46.71 (26.10) | 60.39 (29.99) | 49.07 (24.01) | |
| *p* value | 0.211 | 0.447 | 0.069 | 0.482 | 0.176 | 0.118 | 0.067 | 0.444 | 0.126 | |

*Values presented in this table are means and standard deviations.

* For Paternal and Maternal educational level, Low means Middle school or lower, Middle means High school educated, High means college and higher.

**Supplementary Table S4. Pairwise difference in PedsQL 4.0 GCM scales and total score (n=101)**

|  | Physical functioning | Emotional functioning | Social functioning | School functioning | Total score |
| --- | --- | --- | --- | --- | --- |
| Paternal educational level |  |  |  |  |  |
| Middle vs Low | 14.12 | 11.16 | 8.17 | 8.29 | 11.16 |
| High vs Low | 13.28 | 18.19* | 9.20 | 13.25 | 13.17* |
| High vs Middle | -0.84 | 7.03 | 1.03 | 4.96 | 2.02 |
| Maternal educational level |  |  |  |  |  |
| Middle vs Low | 23.09* | 16.98 | -2.77 | 8.69 | 12.75 |
| High vs Low | 14.91* | 11.07 | -4.86 | 7.66 | 8.16 |
| High vs Middle | -8.18 | -5.91 | -2.08 | -1.03 | -4.59 |
| Having siblings |  |  |  |  |  |
| Yes vs No | -0.73 | 8.61* | 4.16 | 2.30 | 2.79 |
| Annual household income (US dollars) |  |  |  |  |  |
| 14,500 to 36,300 vs Less than 14,500 | 8.21 | 5.53 | -0.74 | 1.41 | 4.17 |
| 36,300 to 72,700 vs 14,500 to 36,300 | 7.09 | 13.19 | 9.17 | 11.37 | 9.74 |
| More than 72,700 vs 14,500 to 36,300 | 18.85* | 17.54 | 7.05 | 9.36 | 14.03 |
| 36,300 to 72,700 vs 14,500 to 36,300 | -1.11 | 7.66 | 9.91 | 9.96 | 5.57 |
| More than 72,700 vs 14,500 to 36,300 | 10.64 | 12.01 | 7.79 | 7.95 | 9.86 |
| More than 72,700 vs 36,300 to 72,700 | 11.76 | 4.35 | -2.12 | -2.01 | 4.29 |
| Receiving social benefits/bonuses/subsidies |  |  |  |  |  |
| Yes vs No | -1.54 | -3.47 | -16.7*** | -2.44 | -5.81 |
| Perceived financial burden |  |  |  |  |  |
| Affordable vs Easily Affordable | -14.22 | -7.99 | -4.53 | -10.60 | -9.79 |
| Hard to afford vs Easily Affordable | -25.83*** | -18.75 | -14.71 | -12.73 | -19.30* |
| Hard to afford vs Affordable | -11.61* | -10.76 | -10.17 | -2.14 | -9.51* |
| School type |  |  |  |  |  |
| Special Education School or Not go to school yet | -0.96 | -3.42 | -6.83 | -16.18* | -5.00 |
| Not go to school yet vs Normal school | -3.59 | 0.48 | 4.98 | -27.93*** | -3.45 |
| Others vs Normal school | -11.76 | -13.76 | 0.75 | -22.54* | -10.25 |
| Not go to school yet vs Special Education School or Recovery Education School | -2.63 | 3.89 | 11.81 | -11.74 | 1.55 |
| Others vs Special Education School or Recovery Education School | -10.81 | -10.34 | 7.58 | -6.36 | -5.26 |
| Others vs Not go to school yet | -8.17 | -14.23 | -4.23 | 5.38 | -6.81 |
| Attention deficit and hyperactivity disorder symptoms |  |  |  |  |  |
| Yes vs No | 0.57 | -0.47 | -9.61* | 6.40 | -1.17 |
| Sleeping problems |  |  |  |  |  |
| Yes vs No | -10.45* | -12.46** | -4.48 | -13.67* | -9.68** |

a: Values presented in this table is difference between two groups.

b: p<0.05 is displayed as *, p<0.01 is displayed as **, p<0.001 is displayed as ***.

c: For Paternal and Maternal educational level, Low means Middle school or lower, Middle means High school educated, High means college and higher.

**Supplementary Table S5. Effect sizes for subgroup differences of PedsQL 4.0 GCM scales and total scores (n=101)**

|  | Physical functioning | Emotional functioning | Social functioning | School functioning | Total score |
| --- | --- | --- | --- | --- | --- |
| Paternal educational level |  |  |  |  |  |
| Low vs. middle | 0.63 | 0.45 | 0.36 | 0.31 | 0.62 |
| Low vs. high | 0.60 | 0.88 | 0.41 | 0.50 | 0.82 |
| Middle vs. high | 0.04 | 0.28 | 0.05 | 0.19 | 0.11 |
| Annual household income (US dollars) |  |  |  |  |  |
| Less than 14,500 vs. 14,500 to 36,300 | 0.37 | 0.25 | 0.03 | 0.05 | 0.26 |
| Less than 14,500 vs. 36,300 to 72,700 | 0.30 | 0.59 | 0.39 | 0.38 | 0.49 |
| Less than 14,500 vs. more than 72,700 | 0.86 | 0.78 | 0.30 | 0.35 | 0.87 |
| 14,500 to 36,300 vs. 36,300 to 72,700 | 0.05 | 0.37 | 0.42 | 0.34 | 0.28 |
| 14,500 to 36,300 vs. more than 72,700 | 0.53 | 0.56 | 0.37 | 0.31 | 0.65 |
| 36,300 to 72,700 vs. more than 72,700 | 0.49 | 0.20 | 0.09 | 0.07 | 0.22 |
| Perceived financial burden |  |  |  |  |  |
| Easily Affordable vs. affordable | 0.72 | 0.40 | 0.21 | 0.39 | 0.60 |
| Easily Affordable vs. hard to afford | 1.32 | 0.80 | 0.63 | 0.50 | 1.23 |
| Affordable vs. hard to afford | 0.59 | 0.46 | 0.44 | 0.08 | 0.58 |
| School type |  |  |  |  |  |
| Normal school vs. special school/recovery school | 0.04 | 0.15 | 0.28 | 0.61 | 0.27 |
| Normal school vs. not go to school yet | 0.19 | 0.02 | 0.22 | 1.07 | 0.22 |
| Normal school vs. others | 0.46 | 0.57 | 0.03 | 0.71 | 0.57 |
| Special school/recovery school vs. not go to school yet | 0.11 | 0.17 | 0.48 | 0.44 | 0.08 |
| Special school/recovery school vs. others | 0.42 | 0.43 | 0.31 | 0.20 | 0.28 |
| Not go to school yet vs. others | 0.32 | 0.58 | 0.19 | 0.17 | 0.38 |
| Presence of attention deficit and hyperactivity |  |  |  |  |  |
| No vs. yes | 0.03 | 0.02 | 0.41 | 0.23 | 0.07 |
| Sleeping problems |  |  |  |  |  |
| No vs. yes | 0.46 | 0.54 | 0.20 | 0.47 | 0.53 |

**Supplementary Table S6. Means and standard deviations of PedsQL 4.0 GCM total scores and scale scores for non-significant variables (n=101)**

|  | Physical functioning | Emotional functioning | Social functioning | School functioning | Total score |
| --- | --- | --- | --- | --- | --- |
| Gender |  |  |  |  |  |
| Boy (N=66) | 59.90 (20.05) | 57.73 (21.43) | 37.05 (23.45) | 37.12 (26.04) | 50.23 (16.90) |
| Girl (N=35) | 58.93 (22.14) | 57.86 (22.66) | 45.29 (19.29) | 36.24 (27.66) | 51.78 (16.18) |
| *p* value | 0.824 | 0.977 | 0.078 | 0.874 | 0.658 |
| Parental marital status | | |  |  |  |
| Married (N=96) | 59.67 (21.03) | 58.23 (21.72) | 40.26 (22.03) | 37.01 (26.70) | 51.07 (16.93) |
| Divorced or Widowed (N=5) | 57.50 (13.73) | 49.00 (23.02) | 33.00 (29.92) | 33.00 (23.87) | 45.00 (5.57) |
| *p* value | 0.821 | 0.358 | 0.482 | 0.743 | 0.428 |
| Living together | |  |  |  |  |
| Always (N=87) | 59.91 (20.75) | 58.33 (21.76) | 40.69 (22.61) | 39.23 (26.24) | 51.62 (16.67) |
| Sometimes (N=10) | 52.50 (19.70) | 53.50 (21.35) | 34.00 (18.53) | 22.50 (23.03) | 43.57 (17.24) |
| Never (N=4) | 69.53 (21.40) | 56.25 (27.50) | 37.50 (28.72) | 20.00 (28.28) | 50.31 (11.20) |
| *p* value | 0.350 | 0.797 | 0.658 | 0.071 | 0.351 |
| Numbers of family members living in the same household | | |  |  |  |
| Three or less (N=19) | 57.24 (12.80) | 48.95 (20.32) | 36.84 (25.01) | 36.93 (22.70) | 47.03 (12.26) |
| Four (N=35) | 63.84 (22.09) | 61.86 (19.52) | 38.29 (23.61) | 39.05 (26.58) | 53.30 (17.47) |
| Five (N=25) | 55.38 (20.76) | 56.20 (22.88) | 45.60 (22.09) | 32.67 (31.06) | 49.72 (17.49) |
| Six and more (N=22) | 59.52 (23.65) | 60.68 (23.97) | 38.64 (18.14) | 37.88 (24.89) | 51.16 (17.71) |
| *p* value | 0.435 | 0.181 | 0.531 | 0.83 | 0.601 |
| Residence | |  |  |  |  |
| Urban (N=66) | 62.03 (19.71) | 60.68 (21.41) | 40.45 (22.08) | 39.37 (26.33) | 52.88 (15.10) |
| Town or Rural (N=35) | 54.91 (21.97) | 52.29 (21.64) | 38.86 (23.14) | 32.00 (26.45) | 46.79 (18.68) |
| *p* value | 0.100 | 0.065 | 0.734 | 0.185 | 0.079 |
| Health Insurance |  |  |  |  |  |
| No (N=21) | 57.44 (22.09) | 59.52 (21.50) | 42.38 (21.94) | 42.38 (28.11) | 52.28 (16.52) |
| Yes (N=80) | 60.12 (20.42) | 57.31 (21.93) | 39.25 (22.55) | 35.35 (26.02) | 50.37 (16.69) |
| *p* value | 0.600 | 0.681 | 0.570 | 0.281 | 0.642 |
| Cost related to WS treatment per year (US dollars) | | |  |  |  |
| Less than 14,500 (N=91) | 59.24 (20.31) | 58.85 (20.32) | 40.71 (21.94) | 36.39 (26.84) | 51.01 (16.35) |
| 14,500 to 32,300 (N=10) | 62.50 (24.91) | 48.00 (31.82) | 32.50 (25.85) | 40.67 (23.74) | 48.58 (19.53) |
| *p* value | 0.638 | 0.135 | 0.272 | 0.630 | 0.662 |
| Having a genetic test | |  |  |  |  |
| No (N=10) | 58.12 (19.50) | 49.50 (20.74) | 42.50 (22.88) | 34.33 (21.95) | 48.99 (12.27) |
| Yes (N=91) | 59.72 (20.91) | 58.68 (21.78) | 39.62 (22.40) | 37.09 (27.02) | 50.97 (17.04) |
| *p* value | 0.819 | 0.207 | 0.700 | 0.757 | 0.722 |
| Getting rehabilitation | |  |  |  |  |
| No (N=41) | 57.77 (19.95) | 55.24 (20.73) | 41.22 (20.61) | 31.46 (24.14) | 48.98 (13.69) |
| Yes (N=60) | 60.78 (21.26) | 59.50 (22.43) | 39.00 (23.59) | 40.47 (27.56) | 51.99 (18.33) |
| *p* value | 0.476 | 0.337 | 0.626 | 0.093 | 0.373 |
| Diseases at birth | |  |  |  |  |
| No (N=23) | 59.51 (19.41) | 51.96 (24.81) | 36.30 (22.72) | 40.14 (28.49) | 49.16 (16.04) |
| Yes (N=78) | 59.58 (21.17) | 59.49 (20.63) | 40.96 (22.27) | 35.83 (25.96) | 51.25 (16.82) |
| *p* value | 0.990 | 0.145 | 0.382 | 0.495 | 0.599 |
| Congenital heart disease and surgery | | | |  |  |
| No (N=18) | 63.37 (22.98) | 62.22 (26.75) | 43.33 (20.58) | 45.09 (23.58) | 55.01 (17.60) |
| With CHD but no surgery (N=63) | 59.38 (20.46) | 58.02 (20.74) | 40.24 (22.83) | 35.24 (26.95) | 50.69 (16.12) |
| With CHD after surgery (N=20) | 56.72 (19.80) | 53.00 (20.09) | 35.75 (22.78) | 34.33 (27.24) | 47.21 (17.14) |
| *p* value | 0.614 | 0.427 | 0.573 | 0.343 | 0.354 |
| Visual system conditions | | |  |  |  |
| Normal (N=17) | 65.26 (17.64) | 57.35 (26.82) | 38.82 (21.83) | 41.86 (24.71) | 53.33 (15.34) |
| Abnormal (N=29) | 54.31 (19.00) | 53.45 (18.03) | 34.48 (21.31) | 36.32 (21.92) | 46.22 (15.48) |
| Not tested (N=55) | 60.57 (22.08) | 60.18 (21.88) | 43.09 (22.84) | 35.52 (29.29) | 52.38 (17.33) |
| *p* value | 0.194 | 0.405 | 0.241 | 0.688 | 0.214 |
| Endocrinologic system conditions | | | |  |  |
| No (N=44) | 60.87 (21.14) | 59.32 (21.66) | 37.50 (22.45) | 41.82 (26.05) | 51.78 (17.06) |
| Yes (N=57) | 58.55 (20.47) | 56.58 (21.94) | 41.75 (22.29) | 32.95 (26.38) | 49.99 (16.33) |
| *p* value | 0.580 | 0.533 | 0.345 | 0.095 | 0.592 |
| Inguinal hernia | |  |  |  |  |
| No (N=51) | 58.58 (18.78) | 57.35 (21.03) | 36.86 (21.21) | 35.03 (25.25) | 49.36 (14.60) |
| Yes (N=28) | 63.06 (22.08) | 60.00 (20.55) | 40.54 (23.27) | 41.31 (25.15) | 53.19 (18.34) |
| Not tested (N=22) | 57.39 (23.42) | 55.91 (25.43) | 46.14 (23.40) | 35.23 (31.14) | 50.97 (18.94) |
| *p* value | 0.565 | 0.792 | 0.265 | 0.577 | 0.621 |
| Repeated respiratory tract infection | | | |  |  |
| No (N=77) | 59.29 (20.45) | 57.27 (21.14) | 41.10 (23.09) | 35.89 (25.79) | 50.60 (16.38) |
| Yes (N=24) | 60.42 (21.88) | 59.38 (24.02) | 36.04 (19.73) | 39.79 (28.94) | 51.31 (17.62) |
| *p* value | 0.818 | 0.681 | 0.335 | 0.531 | 0.856 |
| Digestive system conditions | | |  |  |  |
| No (N=76) | 61.10 (20.04) | 57.83 (22.51) | 40.00 (23.28) | 36.89 (27.50) | 51.37 (16.85) |
| Yes (N=25) | 54.88 (22.32) | 57.60 (19.69) | 39.60 (19.68) | 36.60 (23.61) | 48.94 (15.98) |
| *p* value | 0.193 | 0.964 | 0.939 | 0.963 | 0.529 |
| Urinary system conditions | | |  |  |  |
| No (N=55) | 63.35 (19.26) | 61.91 (19.01) | 39.36 (23.63) | 38.73 (27.61) | 53.30 (15.86) |
| Yes (N=8) | 62.50 (28.40) | 59.38 (25.13) | 46.25 (21.51) | 37.50 (26.05) | 53.64 (20.37) |
| Not tested (N=38) | 53.45 (20.04) | 51.45 (23.76) | 39.34 (20.93) | 33.90 (25.28) | 46.50 (16.39) |
| *p* value | 0.069 | 0.071 | 0.708 | 0.691 | 0.132 |

*Values presented in this table are means and standard deviations.

* For Paternal and Maternal educational level, Low means Middle school or lower, Middle means High school educated, High means college and higher.
